# Adherence to HEI-2010 and odds of breast cancer according to the menopause status: Evidence from Middle Eastern Country

**DOI:** 10.1371/journal.pone.0300986

**Published:** 2024-03-28

**Authors:** Soraiya Ebrahimpour-Koujan, Sanaz Benisi-Kohansal, Leila Azadbakht, Maryam Fallah, Ahmad Esmaillzadeh

**Affiliations:** 1 Department of Clinical Nutrition, School of Nutritional Sciences and Dietetics, Tehran University of Medical Sciences, Tehran, Iran; 2 Department of Community Nutrition, School of Nutritional Sciences and Dietetics, Tehran University of Medical Sciences, Tehran, Iran; Tabriz University of Medical Sciences, ISLAMIC REPUBLIC OF IRAN

## Abstract

**Background:**

Majority of earlier studies have assessed the association between individual healthy eating index-2010 (HEI-2010) and the odds of breast cancer (BC). However, no study has been conducted on the effect of compliance with HEI-2010 and the odds of BC in the Iranian population with a large sample size. Therefore, we aimed to investigate the relationship between the HEI-2010 and the odds of BC in the Iranian population.

**Method:**

This population-based case-control study included 350 newly diagnosed cases of BC and 700 healthy controls randomly selected from adult women. HEI-2010 was examined using validated questionnaires. The adherence to HEI-2010 among the participants was divided into four categories. The general characteristics of the participants in the quartiles of the HEI score for categorical variables and continuous variables were evaluated using chi-square and one-way analysis of variance, respectively. Also, using logistic regression analysis, dietary intakes were evaluated in HEI score quartiles. Also, confounding variables were adjusted in different models.

**Result:**

People with the highest HEI score had 60% lower odds of BC (OR: 0.40; 95% CI: 0.27, 0.57) than those with the lowest score among post-menopause women. After controlling for age and energy intake, individuals with the highest HEI score were 78% less likely to have BC compared with those with the lowest score (OR: 0.22; 95% CI: 0.14, 0.33). Adjustments for other potential confounders including demographic factors made the association stronger (OR: 0.21; 95% CI: 0.13, 0.32). This association remained significant even after taking BMI into model (OR: 0.27; 95% CI: 0.17, 0.43).

**Conclusion:**

Finally, in this study we found an association between HEI-2010 and odds of breast cancer. This association was particularly seen in postmenopausal women. No significant association was found between adherence to HEI-2010 and odds of BC among pre-menopausal.

## Introduction

Breast cancer (BC) is the most common malignancy in women that threating their health in both developing and developed countries [1,2]. There are approximately 464,000 new cases of breast cancer (29% of all new cancers) in Europe and 131,000 breast cancer specific deaths, which accounts for 17% of all cancer deaths [3]. Earlier studies in Iran have estimated that the prevalence of breast cancer was 120 per 100,000 among adult women [4]. It accounts for more than 20% of all cancers in Iran [5].

Several dietary and non-dietary risk factors for BC have been identified. In terms of diet, prior studies have shown that high intake of foods containing W_3_ polysaturated fatty acid (PUFA), vitamin D, phytoestrogens, fiber and folate along with lower intakes of saturated fats, W_6_ PUFA, grilled meat and alcohol might be beneficial in preventing BC [6]. Overall, poor diet quality has been suggested as a main risk factor for BC [7]. To assess diet quality, Healthy Eating Index (HEI) had been developed by the USDA, and was then updated based on the Food Guide Pyramid and Dietary Guidelines [8,9]. The original HEI-2010 was consists of 12 components including nine adequacy components [whole fruit, total fruit, whole grains, dairy, total protein foods, seafood & plant proteins, greens & beans, total vegetables, fatty acids] and three moderation component [refined grains, sodium, empty calories] [10,11]. This dietary index reflects the overall diet quality and it is not targeting food choices and macronutrient sources in relation to the risk of chronic diseases [12,13]. Limited information exists linking HEI and AHEI with risk of breast cancer. The majority of pervious investigations indicated the association of these indicators with reduced risk of all-cause mortality, cardiovascular disease and cancer mortality [14,15]. One epidemiologic study in Brazilian adults indicated an inverse association between HEI and risk of mortality from breast cancer [16]. A most recent study from Iran has reported an inverse association between HEI and BC [17]. Also, this study reached significant findings only in pre-menopausal women [17]. However, their sample sizes were very small and can conflict their results and reliability.

We are aware that there are limited and controversial evidences from developing countries on the association between HEI and odds of breast cancer. As lifestyle patterns of people in developing courtiers, especially Middle Eastern people, are different from those in western countries, assessing diet-disease relations in this part of the world is interesting. In particular, dietary intakes of Middle East population have their own characteristics including large intake of carbohydrates, mostly from refined grains, high intakes of Trans fats and SFAs and low consumption of fruits and vegetables along with lack of alcohol intake. Such eating habits might provide some reasons for the high prevalence of breast cancer in these countries. Therefore, the current study aimed to examine the association of HEI with odds of breast cancer in a large sample of Iranian population.

## Materials and methods

### Study population

This project was a population-based case-control study on women aged ≥30 years, who were currently residing in Isfahan, Iran. All cases were diagnosed with BC during the maximum of last 6 months by physical examination and mammography findings. Patients were recruited from among those that referred to hospitals or private clinics in Isfahan, Iran from July 2013 to July 2015. The sample size calculation was based on the type I error of 5% and the study power of 80%. We hypothesized that low Healthy Eating Index score might increase the odds of breast cancer by 1.5 times. Considering the common ratio of 0.25 and the ratio of controls to cases as 2, we reached almost 350 patients with breast cancer and 700 apparently healthy controls. Patients were underwent surgical resection of breast cancer or chemotherapy or radiotherapy or all of them. Breast cancer patients were defined as primary incident breast tumor with invasive behavior which its histology was available from medical registered history. We did not include patients with a history of any type of neoplastic lesion or cysts (exception of current BC) as well as those with a history of any hormone replacement therapy. In addition, those who were on a special diet were not included in this study. Age-matched controls were selected from healthy women, who had no relationship with BC patients or had no family history of breast cancer. In addition to age, we did our best to match controls in terms of socioeconomic status with the cases. Controls met our inclusion criteria (female, Iranian nationally, no history of any malignancy, cysts and medical disorder, having no special diet or hormone replacement therapy) were selected from the general adult population. Eligible subjects including 350 cases and 700 controls were recruited to the study. Written informed consent was obtained from all subjects. The study was ethically approved by the Ethical Committee of Tehran University of Medical Sciences, Tehran, Iran (IR.TUMS.VCR.REC.1397.1036).

### Dietary intake assessment

Dietary data were collected using a106-item Willett-format semi-quantitative dish-based food frequency questionnaire which was designed and validated specifically for Iranian adults [18]. Detailed information about design and validity of this dish-based FFQ was reported elsewhere [19]. In this study, the questionnaires were completed through face-to-face interview by a trained nutritionist. The questionnaire contained five categories of foods and dishes: (1) mixed dishes (cooked or canned, 29 items); (2) carbohydrate-based foods (different types of bread, cakes, biscuits and potato, 10 items); (3) dairy products (dairies, butter and cream, 9 items); (4) fruits and vegetables (22 items); and (5) miscellaneous food items and beverages (including sweets, fast foods, nuts, desserts and beverages, 36 items). Participants were asked to report their dietary intakes of foods and mixed dishes based on nine multiple choice frequency response categories varying from “never or less than once a month” to “12 or more times per day”. The frequency response categories for the food list varied from 6 to 9 choices. For foods consumed infrequently, we omitted the high-frequency categories, while for common foods with a high consumption, the number of multiple-choice categories increased. For instance, the frequency response for tuna consumption included 6 categories, as follows: never or less than once/month, 1–3 times/month, 1 time per week, 2–4 times/week, 5–6 times/week and 1–2 times/day, and for tea consumption, the frequency response included 9 categories, as follows: never or less than 1 cup/ month, 1–3 cups/month, 1–3 cups/week, 4–6 cups/week, 1 cup/day, 2–4 cups/day, 5–7 cups/day, 8–11 cups/day and ≥12 cups/day. Finally, we computed daily intakes of all food items and then converted them to grams per day using household measures [20]. The daily value for each item was calculated according to food composition, average of reported frequency and specified portion size. As for nutrient intakes, it was calculated by the adding together the nutrient contents of all foods and dishes. The nutrient intake for each participant was obtained by Nutritionist IV software which was modified for Iranian foods. Our previous study indicated that this FFQ provided valid and reliable measures of the average long-term dietary intakes [20,21].

### Construction of Eating Index Score (HEI)

With regards to a healthy diet, we used the Healthy Eating Index-2010 (HEI-2010) [22]. The index was composed of 10 components [total and whole fruits, total vegetables, greens and beans, whole grains, dairy, total protein foods, seafood and plant proteins, fatty acids, refined grains, trans fats, sodium] [23]. In the current study, alcohol consumption was not included into the score, due to lack of information in the original dataset. In the construction of index, first we calculated the energy adjusted intakes of the HEI-2010 mentioned components by the residual method [24]. Second, based on the deciles categories of energy adjusted intakes of these components, classification of participants was performed. The usage of decile categories of components instead of quantitative classifications was considered since scoring by deciles would be least disposed to misclassification. Participants in the highest deciles of fruits, vegetables, whole grains, nuts and legumes, long chain omega-3 fats and polyunsaturated fatty acids were given the score of 10, whereas those in the lowest deciles of these items were given the score of 1. Participants in the other deciles of these components were given the corresponding scores. Concerning sugar sweetened drinks and fruit juice, red and processed meat, trans fat, sodium intake, added sugars and saturated fatty acids the lowest deciles were given a score of 10, whereas the highest deciles were given the score of 1. Individuals in deciles 9, 8, 7, 6, 5, 4, 3 and 2 of these components were given the scores of 2, 3, 4, 5, 6, 7, 8 and 9, respectively. Then to calculate the HEI-2010, we summed up the scores for the individual items, resulting in a minimum score of 10 and a maximum score of 100.

### Assessment of breast cancer

All patients with BC were females with newly diagnosed stage I-IV breast cancer from Iranian nationality, for whom in-situ or invasive status of BC was confirmed by physical examination and mammography. Mammography is type of an x-ray imaging used to diagnose breast disease. The harmful side-effect of breast exposure with irradiation by mammography is very low which can be ignored. This imaging method provides a black and white image of breast. For mammography, the patient was placed in a standing, horizontal and vertical position; then breast was compressed for a few seconds between the pages and photography takes place.

### Assessment of other variables

Body weight was measured by a trained nutritionist, without shoes with light clothing using weighing calibrated scale (Seca, Hamburg, Germany) to the nearest 100 g. Height was measured by a mounted tape, without shoes at a standing position near to the wall, using a stadiometer (Seca, Hamburg, Germany) to the nearest 0.5 cm. BMI was calculated through dividing weight in kilograms by height in meters squared. In terms of physical activity, short form of International Physical Activity Questionnaire (IPAQ) was used through face-to-face interviews [25]. All results of the IPAQ were expressed as Metabolic Equivalents-hours per week (MET-h**/**week). A pretested questionnaire was used to collect data on age, marital status, place of residence, education, socio-economic status, history of disease, family history of cancer, breast feeding history, smoking, menopausal status, alcohol use and supplement use.

### Statistical analysis

First, the quartile of HEI-2010 score was calculated to evaluate the association between adherence to HEI-2010 and odds of breast cancer. General characteristics of study participants across quartiles of HEI-2010 score were examined using one-way ANOVA for continues variables and chi-square for categorical variables. Comparison of dietary intakes across quartiles of HEI-2010 score was done using analysis of covariance. The association of HEI-2010 score with odds of breast cancer was assessed by using conditional logistic regression in different models. Age (continuous) and energy intake (continuous) were considered in the first model. Then, we further controlled for residence (rural/urban), marital status (married/not married/other), socio-economic status (poor/middle class/high class), education (educated/not educated), family history of cancer (yes/no), disease history (yes/no), menopausal status (pre-menopause/post-menopause), history of breastfeeding (yes/no), smoking (smoker/non-smoker/ex-smoker), physical activity (continuous) and supplement use (yes/no). BMI (continuous) was taken into account in the final model. In these analyses, the lowest quartile of HEI-2010 score was considered as reference and odds rations in other quartiles were computed. The trend of odds ratios across increasing quartiles of HEI-2010 score was computed through considering the quartiles as an ordinal variable. In addition to the whole study population, the analyses were also done stratified by menopausal status. In these analyses, all above-mentioned covariates were taken into account. All confounders were chosen based on previous publications. The statistical analyses were carried out by using SPSS (version 18). *P* values were considered significant at <0.05.

## Results

Mean age of study participants was 63.7 y and mean BMI was 24.3 kg/m^2^. **Table 1** provides main characteristics of study participants in both case and control groups as well as across quartiles of HEI-2010 score. Patients with breast cancer were more likely to be older and less likely to be married, educated and overweight than controls. Having family history of breast cancer and being post-menopause were highly prevalent among cases than controls. No other significant difference was seen in the distribution of participants in terms of other variables. When we examined across quartiles of HEI-2010 score, individuals in the highest quartile were more likely to be urban residents, married, educated, obese and alcohol user with poor SES compared with those in the lowest quartile. Participants were not significantly different in terms of other variables across quartiles of HEI-2010 score.

**Table 1:** General characteristics of study participants.

	Groups			HEI score quartiles				
	Controls (n = 700)	Cases (n = 350)	P*	1 (n = 254)	2 (n = 277)	3(n = 248)	4(n = 271)	P*
Age (year)	61 ± 10.3	65.3 ± 11.2	< 0.001	62.7 ± 10.9	62.4±10.7	62.0± 11.2	62.7±10.6	0.889
Residing in Urban Region (%)	36.1	36	0.964	23.2	28.5	37.5	54.6	<0.001
Married (%)	88.3	74.6	< 0.001	82.3	77.3	87.5	88.2	0.004
Educated (%)	28.9	17.4	< 0.001	12.6	18.4	27.4	41.3	<0.001
Family History of BC (%)	3.4	9.4	<0.001	7.1	6.9	3.2	4.4	0.149
Smoker (%)	13	17.4	0.055	18.1	14.1	16.1	10.0	0.051
Post-menopause (%)	77.4	88.3	< 0.001	80.7	81.2	79.0	83.0	0.712
Alcohol use (%)	7.4	4.6	0.076	2.4	2.5	6.5	14.1	<0.001
BMI (kg/m ^2^)	25.6 ± 5.1	21.9 ± 4.9	< 0.001	22.5 ± 4.8	23.6 ± 4.9	25.5 ± 5.6	25.6±5.2	<0.001
Physical Activity (MET-hr/wk)	34.9 ± 6.6	35.4 ± 6.7	0.196	35.1 ± 6.7	34.8 ± 6.7	34.6 ± 7.1	35.6 ± 6.0	0.347
Breast Feeding (%)	33.7	34	0.926	33.1	32.9	35.9	33.6	0.883
Poor Social Economic Status (%)	29	33.4	0.311	42.5	35.7	26.2	17.7	< 0.001
History of Disease (%)	8.7	10.3	0.407	9.8	10.8	8.1	8.1	0.623
Supplement Use (%)	10.1	9.4	0.715	11.4	9.0	8.9	10.3	0.743

ǂAll values are Means±SD unless indicates.

* P values were obtained from independent Student’s t test, one-way ANOVA or χ2 test, where appropriate.

Dietary intakes of study participants are presented in **Table 2.** Compared with controls, patients with breast cancer had higher intakes of energy, carbohydrates, total fat, saturated, mono-unsaturated and trans fats, cholesterol, vitamin E, vitamin C, potassium, zinc, iron, magnesium, calcium as well as fruits, dairy, red and processed meats, egg and salt; and lower intakes of poly-unsaturated fats, vegetables and legumes. High HEI-2010 score was associated with higher intakes of total energy, carbohydrate, total protein, total fat, mono-unsaturated and ploy-unsaturated fatty acids, cholesterol, dietary fiber, vitamin A, vitamin E, vitamin C, vitamin B_6_, folate, vitamin B_12_, potassium, calcium, zinc, copper, selenium, iron, magnesium, whole fruit, total vegetable, sea foods, legume, dairy, whole grain, red and processed meat, white meat and egg and lower intake of saturated and trans fatty acids, refined grain and salt.

**Table 2:** Dietary intakes of study participants.

	Groups			HEI score quartiles				
	Controls (n = 700)	Cases (n = 350)		1 (n = 254)	2 (n = 277)	3(n = 248)	4(n = 271)	
	Mean±SD	Mean±SD	P*	Mean±SD	Mean±SD	Mean±SD		P*
Total energy (kcal/d)	2177.6 ± 608.5	2499.6 ± 793.4	< 0.001	2115± 746.2	2184 ± 631.4	2293± 667.0	2540 ± 649.8	<0.001
**Nutrients**								
Carbohydrates(g/day)	305.8 ± 96.8	340 ± 123.1	<0.001	304.9± 115.9	302.9 ± 109.7	314.0± 101.8	346.5±96.2	<0.001
Total Protein(g/day)	77 ± 27.8	78.6 ± 30.3	0.381	60.7 ± 23.1	71.0 ± 21.1	81.0 ± 30.0	96.8 ± 26.7	<0.001
Fats (g/day)	77.6 ± 28.4	98.6 ± 41.9	<0.001	78.2 ± 38.0	82.3 ± 34.8	85.3 ± 29.6	92.3 ± 35.3	<0.001
Saturated fats (g/day)	26.1 ± 19.9	44.1 ± 33.3	<0.001	38.0 ± 27.1	34.8 ± 30.5	31.6 ± 20.3	24.3 ± 24.9	<0.001
Monounsaturated fats (g/day)	19.8 ± 7.7	21.3 ± 9.3	0.011	16.6 ± 8.0	18.7 ± 6.4	21.0 ± 7.6	24.9 ± 8.7	<0.001
Polyunsaturated fats (g/day)	10.5 ± 8.1	9 ± 4.4	<0.001	7.0 ± 3.4	8.5 ± 3.6	10.2 ± 5.8	14.3 ± 10.6	<0.001
Trans FA (g/day)	0.35 ± 0.2	0.55 ± 0.4	<0.001	0.48± 0.32	0.45 ± 0.37	0.41 ± 0.24	0.35±0.30	<0.001
Cholesterol (mg/day)	181.9 ± 97.3	204.8 ± 134.3	0.005	132.0 ± 88.6	168.0 ± 92.1	196.9 ± 101.9	258.7±119.7	<0.001
Total fiber (g/day)	22.2 ± 7.6	22.7 ± 8.4	0.328	18.9± 7.5	20.5 ± 6.8	22.8 ± 6.7	27.2±7.8	<0.001
Vitamin A (mg/day)	3093 ± 2555	3276.8 ± 2940.7	0.297	1779.3± 1297.1	2353.6 ± 1519.3	3200.3 ± 2208.9	5219.2±3604.1	<0.001
Vitamin D (mg/day)	33.5 ± 51.1	52.6 ± 242.1	0.145	41.9± 273.0	24.9 ± 32.1	42.6 ± 88.3	50.8±66.7	<0.206
Vitamin E (mg/day)	6.3 ± 3.5	6.8 ± 3.2	0.008	5.6± 2.7	5.9 ± 2.8	6.5 ± 3.2	7.8 ±4.2	<0.001
Vitamin C (mg/day)	58.2 ± 38.1	69.1 ± 44.8	<0.001	45.0± 32.2	49.1 ± 25.5	62.9 ± 40.0	89.6±46.2	<0.001
Vitamin B_6_ (mg/day)	1.6 ± 0.5	1.6 ± 0.6	0.052	1.3± 0.5	1.4 ± 0.4	1.7 ± 0.5	2.0±0.5	<0.001
Folate(mg/day)	580.4 ± 189	601.2 ± 205.7	0.103	528.9± 197.6	553.8 ± 183.6	589.8± 180.4	674.1±186.7	<0.001
Vitamin B_12_ (mg/day)	2.6 ± 2.1	3 ± 2.8	0.056	2.1± 2.8	2.3 ± 1.4	2.9 ± 2.5	3.8±2.5	<0.001
Potassium (mg/day)	2849.7 ± 817.7	3070.7 ± 1128.4	0.001	2505.8± 949.9	2737.4 ± 834.6	2947.1 ± 843.8	3483.4±833.9	<0.001
Calcium (mg/day)	736.5 ±276.5	821.6 ±330.9	<0.001	616.8±311.6	701.6±250.2	760.0±237.5	972.7±266.5	<0.001
Sodium (mg/day)	4795.3 ± 1711	4978.9± 1867.5	0.123	4871.9± 1843.1	4767.7 ± 1799.3	4818.4 ± 1781.6	4967.8±1642.6	<0.591
Zinc (mg/day)	10 ± 3.1	10.5 ± 3.5	0.017	8.4± 2.8	9.4 ± 2.5	10.5 ± 3.0	12.4±3.1	<0.001
Copper (mg/day)	1.7 ± 0.5	1.7 ± 0.6	0.086	1.5± 0.6	1.6 ± 0.5	1.7 ± 0.5	2.0±0.5	<0.001
Selenium (mg/day)	145.7 ± 52.5	148.1 ± 52.5	0.493	125.9 ± 50.0	136.4± 46.1	150.9 ± 50.1	172.2±52.2	<0.001
Iron (mg/day)	16.7 ± 5.4	17.6 ± 5.7	0.009	15.0 ± 5.4	15.8± 4.5	17.2 ± 5.0	19.9±5.3	<0.001
Magnesium (mg/day)	441.8 ± 141	472 ± 156.4	0.002	400.1 ± 152.3	427.3 ± 134.9	458.0 ± 133.3	519.9±139.7	<0.001
**Food groups**								
Whole Fruit (g/day)	139.7 ± 122.1	215.1 ± 194.7	<0.001	119.3± 139.5	127.3 ± 106.7	168.5 ± 156.8	242.6 ± 175.1	<0.001
Total Vegetables (g/day)	86.9 ± 75	68.9 ± 66.6	<0.001	45.0± 38.2	52.6 ± 31.9	93.6 ± 71.7	131.9 ± 93.1	<0.001
Sea foods (g/day)	6.5 ± 12.3	11.6 ± 51.1	0.066	6.7 ± 53.6	4.2 ± 8.4	7.8 ± 16.7	13.9 ± 26.7	0.002
Legumes (g/day)	15.5 ± 15.9	13.3 ± 12.9	0.032	8.2 ± 7.2	13.3 ± 10.9	14.4 ± 10.5	14.4 ± 10.5	<0.001
Dairy (g/day)	218.6 ± 143.2	257.5 ± 174.5	<0.001	172.8 ± 174.2	215.3 ± 139.7	226.8 ± 129.9	307.6 ±143.6	<0.001
Refined grains (g/day)	114.6 ± 74.4	115.4 ± 85.9	0.887	126.9 ± 85.0	109.0 ± 73.2	107.6 ± 66.6	116.2 ±84.8	0.021
Whole grains (g/day)	312.6 ± 156.8	325.5 ± 150.2	0.197	289.4 ± 159.2	301.7 ± 147.7	326.6 ± 148.4	349.2 ±157.2	<0.001
Red and processed meat (g/day)	9.6 ± 12.4	13.3 ± 20.1	0.002	7.9 ± 10.2	9.8 ± 14.1	11.5 ± 17.9	14.1 ±17.9	<0.001
White meat (g/day)	74.1 ± 67.6	70.6 ± 89.9	0.478	43.3 ± 66.6	63.9 ± 57.8	81.6 ± 91.7	102.0 ± 72.2	<0.001
Egg (g/day)	10.4 ± 12.4	12.6 ± 19	0.050	8.2± 13.5	9.8 ± 15.2	10.7 ± 11.8	15.6 ±17.5	<0.001
Salt (g/day)	2.7 ± 1.7	3.5 ± 3.5	<0.001	3.7 ± 3.2	3.2 ± 2.7	2.6 ± 1.7	2.2 ±1.5	<0.001

*Obtained by ANOVA.

Crude and multivariable-adjusted ORs for breast cancer across quartiles of healthy eating index 2010 (HEI-2010) score are shown in **Table 3**. In the whole study population, those with the highest HEI score had significantly lower odds of BC (OR: 0.40; 95% CI: 0.27, 0.57) than those with the lowest score. After controlling for age and energy intake, individuals with the highest HEI score were 78% less likely to have BC compared with those with the lowest score (OR: 0.22; 95% CI: 0.14, 0.33). Adjustments for other potential confounders including demographic factors made the association stronger (OR: 0.21; 95% CI: 0.13, 0.32). This association remained significant even after taking BMI into model (OR: 0.27; 95% CI: 0.17, 0.43).

**Table 3:** Multivariable-adjusted ratios for BC across different quartiles of HEI score.

	1 (n = 254) OR	2 (n = 277) OR 95% CI		3 (n = 248) OR 95% CI		4 (n = 271)		P trend
						OR 95% CI		
**All population**								
**HEI score ranges**	**<58**	**58–65**		**65–74**		**74<**		
**Crude**	1.00	0.60	0.42–0.85	0.25	0.17–0.37	0.40	0.27–0.57	<0.001
**Model I**	1.00	0.52	0.36–0.76	0.17	0.11–0.27	0.22	0.14–0.33	<0.001
**Model II**	1.00	0.47	0.32–0.70	0.17	0.11–0.27	0.21	0.13–0.32	<0.001
**Model III**	1.00	0.49	0.32–0.74	0.22	0.13–0.35	0.27	0.17–0.43	<0.001
**Pre-menopause**								
**HEI score ranges**	**<58**	**58–65**		**65–72**		**72<**		
**Crude**	1.00	0.16	0.50–0.51	0.63	0.26–1.50	0.12	0.12–0.91	0.129
**Model I**	1.00	0.14	0.04–0.48	0.60	0.24–1.49	0.23	0.08–0.67	0.044
Model II*	1.00	0.12	0.03–0.46	0.77	0.24–2.14	0.25	0.07–0.90	0.133
**Model III**	1.00	0.12	0.03–0.55	1.93	0.49–7.64	1.43	0.26–7.95	0.309
**Post-menopause**								
**HEI score ranges**	**<58**	**58–65**		**65–74**		**74<**		
**Crude**	1.00	0.69	0.47–1.01	0.19	0.12–0.31	0.39	0.26–0.56	<0.001
**Model I**	1.00	0.60	0.40–0.90	0.12	0.07–0.20	0.20	0.13–0.32	<0.001
Model II*	1.00	0.53	0.35–0.82	0.11	0.06–0.19	0.18	0.11–0.30	<0.001
**Model III**	1.00	0.54	0.34–0.85	0.13	0.08–0.23	0.22	0.13–0.37	<0.001

Model I: Adjusted for age and energy.

Model II: Additional adjustment for residence, marital status, SES, education, family history of B.C, history of disease, menopausal status, breast feeding, smoking, physical activity and supplement use.

Model III: Further adjustment for BMI.

Model II*: Residence, marital status, SES, education, family history of B.C, history of disease, breast feeding, smoking, physical activity and supplement use.

Stratified-analysis by menopausal status revealed that pre-menopausal women with the highest HEI had 82% lower odds of BC compared with those with the lowest score (OR: 0.12; 95% CI: 0.12, 0.91). This association was significant even after taking other confounders in the model (OR: 0.25; 95% CI: 0.07, 0.90). However, adjustment for BMI made the association non-significant (OR: 1.43; 95% CI: 0.26, 7.95). By stratified analysis, we found that post-menopausal women in the top quartile of HEI were 61% less likely to have breast cancer compared to those in the bottom quartile (OR: 0.39; 95% CI: 0.26, 0.56). Taking other confounding factors including age, energy intake and demographic factors made the association stronger (OR: 0.18; 95% CI: 0.11, 0.30). Further controlling for BMI showed that post-menopausal women in the highest quartile of HEI were 78% less likely to have breast cancer compared to those in the first quartile (OR: 0.22; 95% CI: 0.13, 0.37).

## Discussion

In this large population-based case-control study, we found a significant inverse association between higher healthy eating index-2010 (HEI-2010) score and reduced odds of breast cancer among Iranian whole population as well as post-menopausal women. These associations persisted in multivariate models even after adjustment for potential confounders including demographic, life-style related factors and BMI. However, there were no significant association between adherence to HEI-2010 and odds of breast cancer among pre-menopauses.

Breast cancer is among most prevalent cancers worldwide, in particular among Iranian women whom their age of breast cancer initiation is lower than other parts, as well [26,27]. In common, network of risk factors including environmental and genetic indictors were involved in breast cancer incidence [28]. However, the modifiable variables in particular diet-related factors are more paid attention which are justified 25–30% of causality [29,30]. Modifications in healthy eating that reflects diet quality were frequently considered for breast cancer prevention [31,32]. In the current study, we found that adhering to HEI-2010 was associated with a reduced odds of BC especially in post-menopausal women. These results were in line with previous reports in which higher scores of HEI were in relation to lower risk of breast cancer risk in whole population [17,33,34]. Despite, Shahril *et al*. have reported inverse association among adherence to HEI-2005 and BC risk in Malaysian women, the ability of HEI-2005 in breast cancer risk prediction is poor and local index-based dietary patterns are needed in this regard [35]. In contrast our findings, some studies have not reached a significant association between HEI-2010 and breast cancer, which may be due to the diversity in food composition, it is not possible to include all food exposures and bias in the self-report [31,36]. When we analyzed data stratified by menopausal status, we found significant association only in post-menopausal. The same findings were shown in a study [37]. In contrast, a small case-control study by Sedaghat *et al*. in Iran has shown a significant association only in among pre-menopausal women [17]. This inconsistent finding may be due to the fact that consumption of total n-3 polyunsaturated fatty acids (PUFA) and soy is negatively associated with atypia in premenopausal women. Low level of docosahexaenoic acid in breast fat leads to atypia in premenopausal period [17]. Furthermore, in a case-control study in Malaysian, inverse relation between higher score of HEI-2005 and lower risk of breast cancer among pre-menopausal women was seen [35]. One of the reasons for the discrepancies in the findings may be due to the difference in the evaluation of various components of HEI, such as alcohol consumption, which was not evaluated in our study [35,38]. In case of controversial findings causes, the most important reason is related to the construction HEI by different versions. We used HEI-2010 version, but some studies have used primary version of HEI-2005 and some other HEI-2015. Therefore, one might question why we used HEI-2010 and did not consider newest version. Earlier studies on HEI have mostly used the version we used in present study. Moreover, alcohol consumption was not considered in our HEI-2010 score due to cultural and religion issues, alcohol consumption is forbidden in our study population. One of important cause of controversial findings is the study population differences in sample size. Our study is a large case-control and its findings are more reliable than other small studies. Finally, lack of significant association among pre-menopausal women in our study might be explained by the low number of pre-menopausal women in the current study (850 post- vs. 200 pre-menopausal women). It is possible that this difference in findings in premenopausal and postmenopausal women is influenced by our study population. In our study, the number of postmenopausal women was more than the number of premenopausal women. This distribution in pre- and post-menopausal patients is different from other studies [16,39]. It is possible that compliance to HEI in postmenopausal is more than premenopausal [37]. Therefore, it may cause to difference in intake of estrogen containing food components. Estrogens are considered to play an important role in increasing the proliferation of normal and neoplastic mammary epithelium [40]. Also, the increase in the level of exogenous hormones as a result of the use of hormone replace therapy (HRT) in postmenopausal women increases the risk of BC [41].

The underlying action mechanism of HEI-2010 against breast neoplasia initiation mostly related to anti-inflammatory property of fruit, vegetable and whole grain rich in bioactive substances and antioxidants [16,42]. As inflammation play major role in BC cell proliferation in particular hormone receptor (ER) negative, a healthy diet that is rich in dietary fiber, antioxidants and vitamins might decrease the BC cell proliferation through suppression of inflammatory cascades, scavenging free radicals and inhibiting DNA damage [43]. A more relevant mechanism is the effects of dietary fiber and other nutrients on reduction of estrogen and N-nitroso compounds that collectively prevent BC initiation and progression [44,45]. Dairy products as source of calcium and vitamin D; and unsaturated fatty acids are considered as anti-cancer agents via binding to cancer-causing acids and metabolites, neutralizing free radicals and alter estrogen metabolism [46,47]. These evidences explain why a diet with high HEI score is associated to lower risk of BC.

This study has several strengths. Our case-control study would be among large sample size studies in Middle Eastern population examining HEI association with odds of BC rather than others. Using validated FFQ, accounting several potential covariates in analyses and stratified analyses by menopausal status could be considered as our study strengths. However, some limitations need to be considered in interpretation of findings. First, one of the reasons why we did not use the new version of HEI is the possibility of errors in the findings. The new version of HEI has a hard construction and due to the use of serving sizes, there was a possibility of errors [48]. Newly published articles have also recommended HEI 2010 and proved that this version of HEI has no limitations in evaluating participants in the studies [49–51]. Second, due to the case-control design of the study which is subject to selection and recall bias, causality cannot be inferred. We did not consider the pathologic differences, in particular estrogen receptor status between various types of breast cancer cases. The hormone receptor differences and their gene expressions might be important mediators of HEI-2010 and its component effects on breast neoplasia, which should be considered in future studies. The use of FFQ for assessment of dietary intakes might result in measurement errors and some sort of misclassification of study participants. It should be explained that HEI-2010 is a diet quality marker based on USA constructed index which might be not appropriate to apply for other different populations and national HEI-2010 index would be more informative.

## Conclusion

In conclusion, we found a protective association between HEI-2010 and odds of breast cancer. This association was particularly seen in postmenopausal women. Therefore, adoption to a healthy diet, especially rich in fruits, vegetables, beans, whole grains, dairy, total protein foods, seafood and plant proteins might help prevent the prevalence of breast cancer in the community setting. No significant association was found between adherence to HEI-2010 and odds of BC among pre-menopausal. Further studies, however, are needed to confirm our current findings.
